# Novel colistin-EDTA combination for successful eradication of colistin-resistant *Klebsiella pneumoniae* catheter-related biofilm infections

**DOI:** 10.1038/s41598-021-01052-5

**Published:** 2021-11-04

**Authors:** Aye Mya Sithu Shein, Dhammika Leshan Wannigama, Paul G. Higgins, Cameron Hurst, Shuichi Abe, Parichart Hongsing, Naphat Chantaravisoot, Thammakorn Saethang, Sirirat Luk-in, Tingting Liao, Sumanee Nilgate, Ubolrat Rirerm, Naris Kueakulpattana, Matchima Laowansiri, Sukrit Srisakul, Netchanok Muhummudaree, Teerasit Techawiwattanaboon, Lin Gan, Chenchen Xu, Rosalyn Kupwiwat, Phatthranit Phattharapornjaroen, Rojrit Rojanathanes, Asada Leelahavanichkul, Tanittha Chatsuwan

**Affiliations:** 1 Department of Microbiology, Faculty of Medicine, Chulalongkorn University, King Chulalongkorn Memorial Hospital, Thai Red Cross Society, Bangkok, Thailand; 2 Antimicrobial Resistance and Stewardship Research Unit, Faculty of Medicine, Chulalongkorn University, Bangkok, Thailand; 3 Interdisciplinary Program of Medical Microbiology, Graduate School, Chulalongkorn University, Bangkok, Thailand; 4 School of Medicine, Faculty of Health and Medical Sciences, The University of Western Australia, Nedlands, WA Australia; 5 Institute for Medical Microbiology, Immunology and Hygiene, Faculty of Medicine and University Hospital Cologne, University of Cologne, Cologne, Germany; 6 Center for Molecular Medicine Cologne, Faculty of Medicine and University Hospital Cologne, University of Cologne, Cologne, Germany; 7 German Centre for Infection Research, Partner Site Bonn-Cologne, Cologne, Germany; 8 Statistics, QIMR Berghofer Medical Research Institute, Brisbane, QLD Australia; 9 Department of Infectious Diseases and Infection Control, Yamagata Prefectural Central Hospital, Yamagata, Japan; 10 Mae Fah Luang University Hospital, Chiang Rai, Thailand; 11 School of Integrative Medicine, Mae Fah Luang University, Chiang Rai, Thailand; 12 Department of Biochemistry, Faculty of Medicine, Chulalongkorn University, Bangkok, Thailand; 13 Center of Excellence in Systems Biology, Research Affairs, Faculty of Medicine, Chulalongkorn University, Bangkok, Thailand; 14 Department of Computer Science, Faculty of Science, Kasetsart University, Bangkok, Thailand; 15 Department of Clinical Microbiology and Applied Technology, Faculty of Medical Technology, Mahidol University, Bangkok, Thailand; 16 Department of Physiology, Faculty of Medicine, Chulalongkorn University, Bangkok, Thailand; 17 Center of Excellence for Microcirculation, Faculty of Medicine, Chulalongkorn University, Bangkok, Thailand; 18 Chula Vaccine Research Center, Faculty of Medicine, Chulalongkorn University, Bangkok, Thailand; 19 Department of General Surgery, Fuling Center Hospital of Chongqing City, Chongqing, China; 20 In-Patient Pharmacy, Jiangsu Province Hospital, The First Affiliated Hospital of Nanjing Medical University, Nanjing, China; 21 Chulabhorn International College of Medicine, Thammasat University, Thammasat University Hospital, Bangkok, Thailand; 22 Department of Emergency Medicine, Center of Excellence, Faculty of Medicine Ramathibodi Hospital, Mahidol University, Bangkok, Thailand; 23 Institute of Clinical Sciences, Department of Surgery, Sahlgrenska Academy, Gothenburg University, 40530 Gothenburg, Sweden; 24 Center of Excellence in Materials and Bio-Interfaces, Faculty of Science, Chulalongkorn University, Bangkok, Thailand; 25 Translational Research in Inflammation and Immunology Research Unit (TRIRU), Department of Microbiology, Chulalongkorn University, Bangkok, Thailand

**Keywords:** Antimicrobials, Bacteria, Bacteriology, Biofilms, Clinical microbiology, Medical research, Infectious diseases

## Abstract

Development of an effective therapy to overcome colistin resistance in *Klebsiella pneumoniae*, a common pathogen causing catheter-related biofilm infections in vascular catheters, has become a serious therapeutic challenge that must be addressed urgently. Although colistin and EDTA have successful roles for eradicating biofilms, no in vitro and in vivo studies have investigated their efficacy in catheter-related biofilm infections of colistin-resistant *K. pneumoniae*. In this study, colistin resistance was significantly reversed in both planktonic and mature biofilms of colistin-resistant *K. pneumoniae* by a combination of colistin (0.25–1 µg/ml) with EDTA (12 mg/ml). This novel colistin-EDTA combination was also demonstrated to have potent efficacy in eradicating colistin-resistant *K. pneumoniae* catheter-related biofilm infections, and eliminating the risk of recurrence in vivo. Furthermore, this study revealed significant therapeutic efficacy of colistin-EDTA combination in reducing bacterial load in internal organs, lowering serum creatinine, and protecting treated mice from mortality. Altered in vivo expression of different virulence genes indicate bacterial adaptive responses to survive in hostile environments under different treatments. According to these data discovered in this study, a novel colistin-EDTA combination provides favorable efficacy and safety for successful eradication of colistin-resistant *K. pneumonia* catheter-related biofilm infections.

## Introduction

*K. pneumoniae* is one of the most prevalent Gram-negative pathogens involved in bacterial colonization and catheter-related biofilm infections which are typical consequences that frequently arise in vascular catheters^1,2^ Bacterial colonization of vascular catheters occurs frequently within 24 h of use and the severity of catheter-related biofilm infections are significantly correlated to the duration of catheterization^2^. Nowadays, occurrence of catheter-related biofilm infections by multidrug-resistant *K. pneumoniae* is increasing^3^, posing a significant challenge in selecting appropriate treatment. Because of rising antibiotic resistance and biofilm tolerance to both antimicrobial and host immunological responses^1^, clinicians are confronted with higher mortality rates^1^, especially in critically ill patients.

Adding to the urgency of the problem, colistin-resistant *K. pneumoniae* infections are increasing in intensive care units, especially in patients who ultimately depend on vascular catheters for short-term (< 10 days) or long-term (≥ 30 days) use, depending on their requirements^4,5^. These catheters are frequently colonised by biofilms which act as a potential source of bloodstream infections during the critical phases when treatment options are limited^1^. In most cases, surgical removal is required, however obtaining alternate venous access for catheter replacement is problematic^6^. Moreover, replacing infected catheters carries the risk of traumatic injuries, limited catheter access, and increased treatment costs^6^. Currently, the antibiotic lock technique can be attempted to control these complications^7^. This procedure targets intraluminal biofilms lining the lumen of the infected catheters through the instillation of a solution containing a high concentration of single or combined antimicrobial agents^7^. Increasing concerns about multi-resistant infections have prompted the evaluation of novel lock solutions that combine different antibiotics or nonantibiotic compounds^7,8^. Ethylenediaminetetraacetic acid (EDTA) is an FDA-approved metal ions chelator with a favorable pharmacokinetic safety profile for intravenous treatment of lead poisoning^9^. EDTA increases outer membrane permeabilization with the release of LPS and augments antibiotic activity by facilitating antibiotic penetration to reach their targets^9^.

EDTA also exhibits antibiofilm activity by disrupting the biofilm matrix via strong metal ions chelation activities^9^. According to previous studies, EDTA increased the effectiveness of existing antibiotics for eradicating Gram-negative bacteria mature biofilms^10–13^. It has preferable bactericidal activities against inner biofilm cells with lower metabolic activities^14^. As colistin monotherapy was unable to eradicate mature biofilms in bacterial infections^15^, we hypothesized that it would be interesting to combine colistin with EDTA, to augment colistin activity, and make it re-effective against resistant pathogens, at lower dosages.

To the best of our knowledge, no study has been conducted to evaluate the activities of novel colistin-EDTA combination on colistin-resistant *K. pneumoniae.* The primary objective of this study was to investigate the effectiveness of novel colistin-EDTA combination in catheter-related biofilm infections of colistin-resistant *K. pneumoniae* both in vitro and in vivo.

## Results

### Colistin-EDTA combination displays synergistic activity against colistin-resistant *K. pneumoniae* clinical isolates

**Figure 1:**
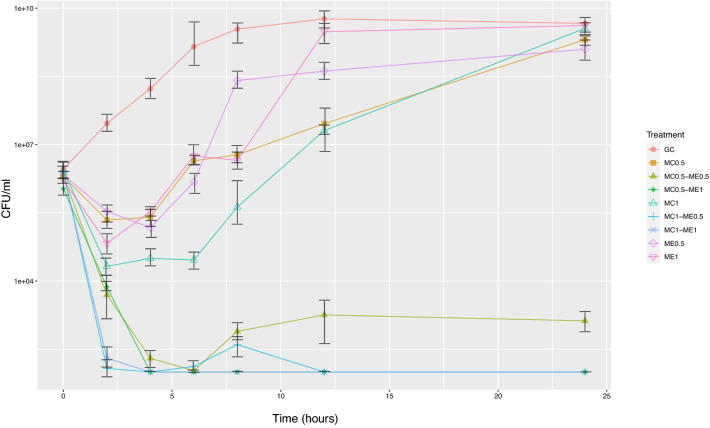
Time-kill assay of pan-drug resistant (PDR) *K. pneumoniae* clinical isolate using colistin, EDTA and colistin-EDTA combination. MC 1(Colistin at 1 × MIC, µg/ml), MC 0.5(Colistin at 0.5xMIC, µg/ml), ME 1 (EDTA at 1xMIC, mg/ml), ME 0.5 (EDTA at 0.5xMIC, mg/ml) in vitro.

All colistin-resistant *K. pneumoniae* clinical isolates had no visible signs of growth in the presence of 3–24 mg/ml EDTA (Table 1). Combination of colistin (0.25 µg/ml) with EDTA (12 mg/ml) exhibited potent synergistic activity (FICI ≤ 0.5) against all planktonic colistin-resistant *K. pneumoniae* isolates (Table 1). Compared to colistin and EDTA alone, colistin-EDTA combination showed ≥ 3 log reduction in CFUs within 2 h (Fig. 1).

**Table 1:** Susceptibilities of planktonic and biofilms of colistin-resistant *Klebsiella pneumoniae* clinical isolates to colistin, EDTA and their combination in vitro.

Specimens	Planktonic				Biofilm		
	Colistin µg/ml	EDTA mg/ml	Colistin µg/ml + EDTA mg/ml		Colistin µg/ml	EDTA mg/ml	Colistin µg/ml + EDTA mg/ml
	MIC^a^	MIC^a^	MIC^a^	FICI^b^	MBEC*	MBEC*	MBEC*
Extensively drug-resistant (XDR) (n = 43)	8– > 2048	3–24	0.25 + 12	0.09375–0.375	16–2048	12–48	0.5 + 12
Pandrug-resistant (PDR) (n = 4)	16–32	12–24	0.25 + 12	0.3125–0.5	64–2048	12–48	1 + 12

Ethylenediaminetetraacetic acid (EDTA).

^a^Minimal inhibitory concentrations (MIC, μgmL^−1^) for planktonic cells.

^b^Fractional inhibition concentration index (FIC index).

*Minimal biofilm eradication concentrations (MBEC, μgmL^−1^) for mature biofilms.

MBEC values for colistin-resistant *K. pneumoniae* biofilms were 8–1000-fold higher for colistin and two–fourfold higher for EDTA alone. Interestingly, colistin (0.5–1 µg/ml) in combination with EDTA (12 mg/ml) was able to completely eradicate the mature biofilms of colistin-resistant *K. pneumoniae* clinical isolates (*p* < 0.05) (Table 1).

### Combination of colistin-EDTA shows potent efficacy in eradicating biofilms within a 24-h treatment exposure

**Figure 2:**
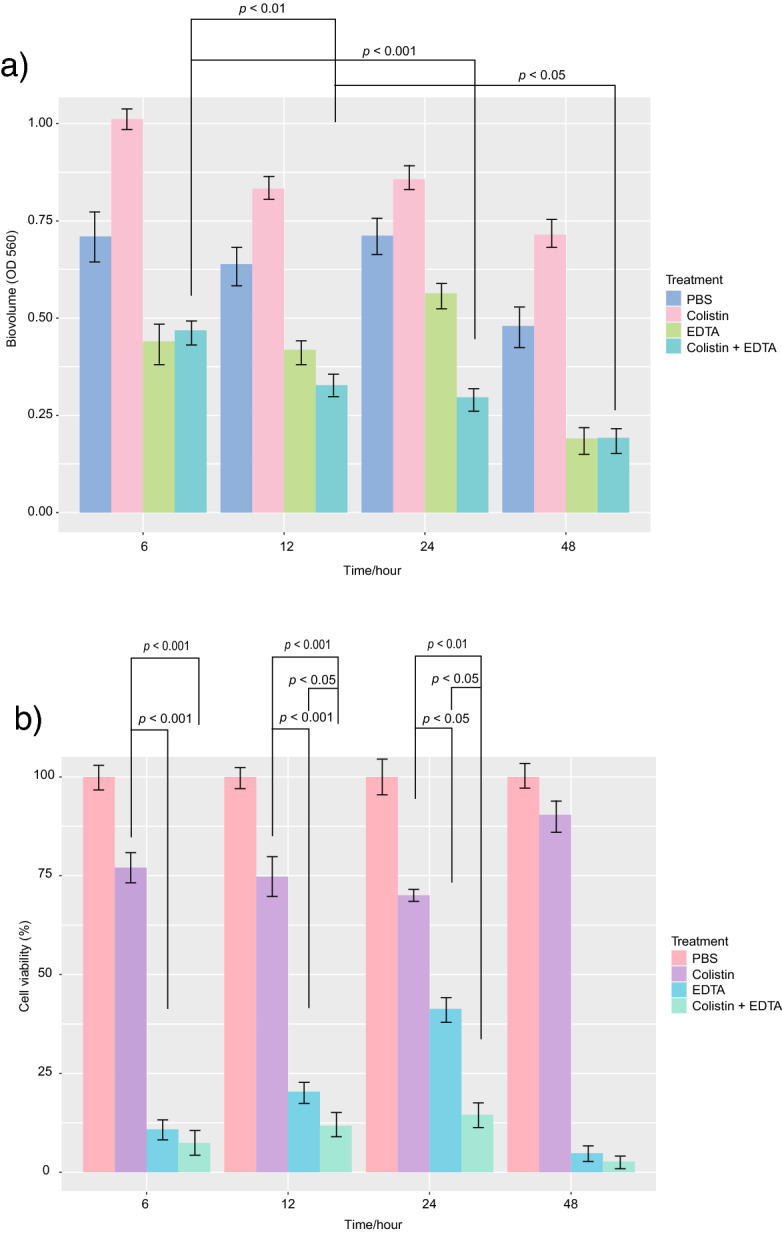
Effects of colistin, EDTA and colistin-EDTA combination on (**a**) biofilm biovolume (OD 560). (**b**) biofilm cell viability (%) of colistin-resistant *K. pneumoniae* at different time interval in vitro. All experiments in (**a**, **b**) were performed as three biologically independent experiments, and the mean ± s.d. is shown. P values were determined using an unpaired, two-tailed Student’s t-test.

The single exposure to colistin (1 µg/ml) plus EDTA (12 mg/ml) in combination displayed progressive reductions in biofilm biovolume in a time-dependent manner with the most pronounced eradication effects within 24 h (*p* < 0.01) (Fig. 2a). Within a short exposure time (6 h), bacterial cell viability within the biofilm was significantly reduced (*p* < 0.001) with colistin-EDTA combination treatment when compared to EDTA or colistin alone (Fig. 2b). When compared to the PBS control (untreated) group, colistin alone resulted in a lower cell viability but a higher rise in biofilm biovolume. Interestingly, EDTA alone displayed significant reduction in bacteria cell viability within the biofilm after 6-h exposure, but sharp increase of viable cells on 12 and 24 h (Fig. 2b).

### Colistin-EDTA combination significantly eradicate catheter-related biofilm infections of colistin-resistant *K. pneumoniae* in vitro and in vivo

**Figure 3:**
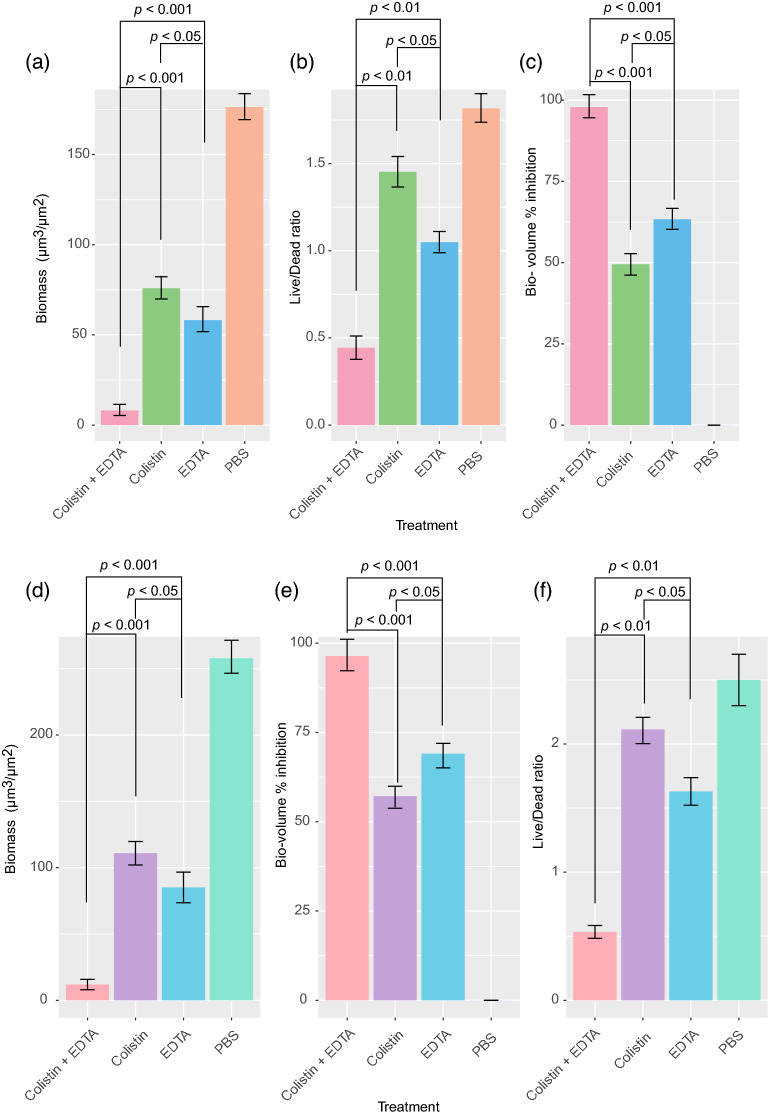
Effects of colistin (1 µg/ml), EDTA (12 mg/ml) and colistin- EDTA combination (1 µg/ml + 12 mg/ml)on colistin-resistant *K. pneumoniae* biofilms (**a**) in vitro biomass (**b**) in vitro Live/Dead cell ratio (**c**) in vitro bio-volume inhibition, and catheter-related biofilm infection of colistin-resistant *K. pneumoniae* (**d**) in vivo biomass, (**e**) in vivo bio-volume inhibition (**f**) in vivo Live/Dead cell ratio. All experiments in (**a**–**f**) were performed as three biologically independent experiments, and the mean ± s.d. is shown. P values were determined using an unpaired, two-tailed Student’s t-test.

**Figure 4:**
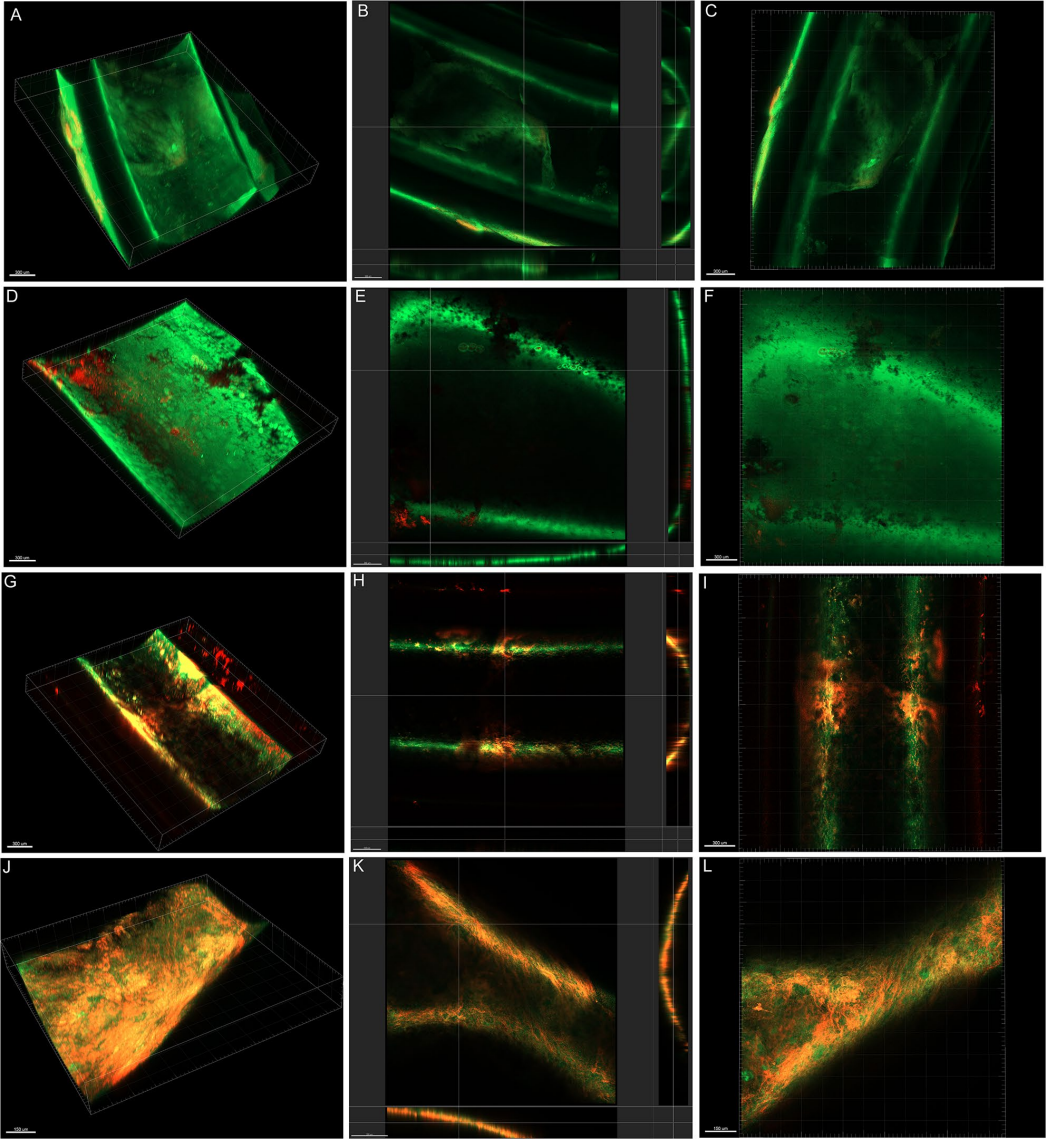
Confocal imaging analysis (3D and cross sectional). (**A**–**C**) PBS-treated, (**D**–**F**) Colistin-treated, (**G**–**I**) EDTA-treated, (**J**–**L**) Colistin-EDTA combination-treated catheter-related biofilm infection of colistin-resistant *K. pneumoniae *in vivo*.* All experiments in (**A**–**L**) were performed as three biologically independent experiments, and the mean ± s.d. is shown. P values were determined using an unpaired, two-tailed Student’s t-test.

There was significant reduction in biofilm biomass (*p* < 0.001) with a significantly lower live/dead ratio (*p* < 0.01) of bacteria within biofilms treated with colistin-EDTA combination (1 µg/ml + 12 mg/ml) when compared to colistin (1 µg/ml) or EDTA (12 mg/ml) alone, both in vitro (Fig. 3a–c) and in vivo (Fig. 3d–f). Furthermore, as compared to colistin and EDTA alone, the colistin-EDTA combination displayed significantly increased inhibitory effects on biofilm biovolume (*p* < 0.001) of colistin-resistant *K. pneumoniae*, both in vitro and in vivo (Fig. 3). Confocal imaging analysis further confirmed the significant reduction in biofilm biovolume and viable cells of colistin-resistant *K. pneumoniae* within biofilms exposed to colistin-EDTA combination, compared to EDTA or colistin alone (Fig. 4).

### Colistin-EDTA combination significantly decreases bacterial load in internal organs and serum creatinine

**Figure 5:**
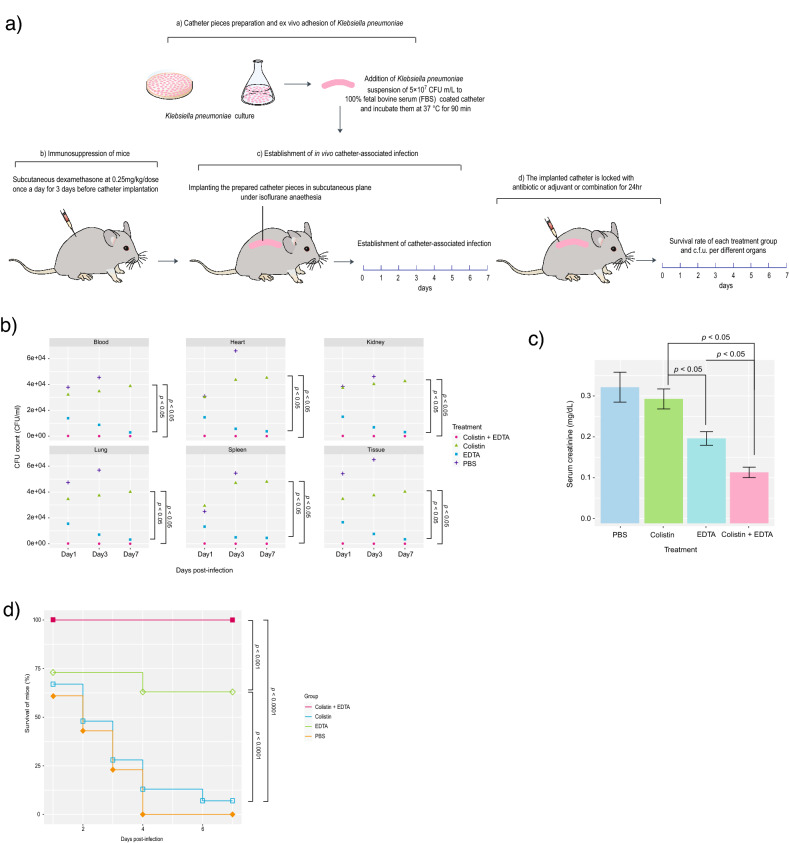
Effects of colistin, EDTA and colistin-EDTA combination on (**a**) illustration of mouse model (**b**) bacterial load in internal organs (**c**) serum creatinine of mice (**d**) survival of mice. *P* values were determined using a two-sided, Mann–Whitney U-test. All data were presented as means ± s.d.

Throughout all tested days, mice treated with colistin-EDTA combination (1 µg/ml + 12 mg/ml) exhibited a significant reduction in *K. pneumoniae* bacterial load in internal organs—blood, heart, kidneys, lungs, spleen, and tissues surrounding catheter, when compared to mice treated with colistin or EDTA alone (*p* < 0.05) (Fig. 5b). Significantly, EDTA-treated mice had lower bacterial loads in various internal organs on all tested days when compared to colistin-treated mice (*p* < 0.05) (Fig. 5b). Furthermore, infected mice treated with colistin-EDTA combination showed significantly lower serum creatinine levels than mice treated with colistin or EDTA alone (*p* < 0.05). In comparison to colistin-treated mice, administration of EDTA alone significantly reduced serum creatinine levels in treated mice (*p* < 0.05) (Fig. 5c).

### Colistin-EDTA combination significantly improves the survival of mice

Mice treated with the colistin-EDTA combination displayed significantly higher survival rates of 100% until day 7 when compared to mice given colistin alone (*p* < 0.0001) or EDTA alone (*p* < 0.001) (Fig. 5d). Interestingly, EDTA-treated mice had significantly higher survival rates than the colistin-treated mice (*p* < 0.0001).

### Exposure to colistin-EDTA combination result in altered expressions of virulent genes in vivo

**Figure 6:**
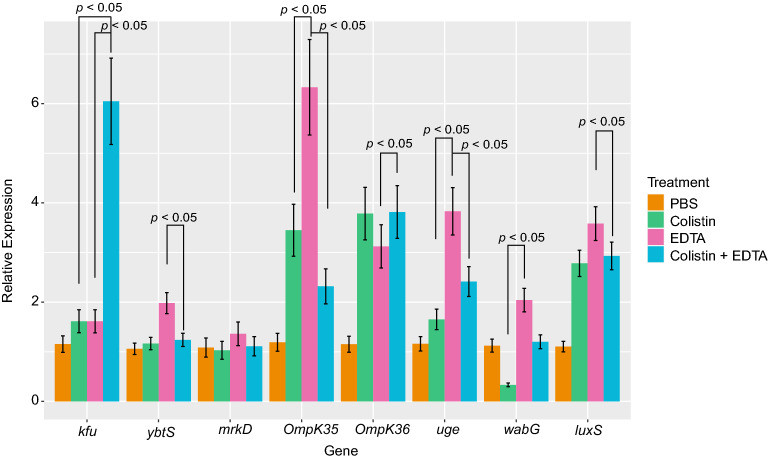
Effect of single and combination of colistin and EDTA on relative expression of virulence genes in vivo.

Expression of *kfu* was significantly increased (*p* < 0.05) after giving colistin-EDTA combination as compared to colistin or EDTA-treated groups. Genes—*ybtS* and *luxS* expression were similar in both colistin and colistin-EDTA-treated groups, however their expression become significantly increased (*p* < 0.05) after EDTA treatment. The expression of *mrkD* was not significantly (*p* > 0.05) affected by all treatments. The gene-*ompK 35* expression was increased significantly (*p* < 0.05) with EDTA as compared to colistin treatment, but the expression level decreased significantly (*p* < 0.05) with colistin-EDTA combination treatment. The expression of *ompK36* was found to be comparable in colistin and colistin-EDTA combination treatments, despite EDTA decreasing expression (*p* < 0.05) of this gene. For *uge* and *wabG*, their expression levels were significantly increased (*p* < 0.05) after exposure to EDTA as compared to colistin and colistin-EDTA combination treatments (Fig. 6).

## Discussion

The rising trend of colistin resistance and catheter-related biofilm infections in *K. pneumoniae* has led to recurrent and often untreatable infections, especially in critically ill patients^3,16^. Therefore, an effective therapy to overcome colistin resistant *K. pneumoniae* catheter-related biofilm infections has become an urgent necessity. In this study, mature colistin-resistant *K. pneumoniae* biofilms showed significant antimicrobial tolerance with an MBEC 1000 times higher than their corresponding MIC values. These findings are in line with a previous study that indicated higher antimicrobial tolerance of mature biofilms due to the barrier effects of biofilm matrix which limit antibiotic penetration^17^. Interestingly, colistin resistance was significantly reversed in both planktonic and mature biofilms of colistin-resistant *K. pneumoniae* when colistin (0.25–1 µg/ml) was given in combination with EDTA (12 mg/ml). These findings agree with a prior study showing synergistic antibacterial effects of antimicrobial peptide AA23 and EDTA on planktonic Gram-negative bacteria^18^.

The enhanced antimicrobial spectrum of colistin-EDTA combination against planktonic colistin-resistant *K. pneumoniae* could be owing to their synergistic interactions in increasing bacterial membrane permeability, which augments intracellular content release, and bacterial death^9,19^. Also, the chelation activities of EDTA may potentiate colistin's entry into bacteria to exert their bactericidal effects by blocking essential respiratory enzymes which are important intracellular targets of colistin^20^.

Increased biofilm biovolume of *K. pneumoniae* following exposure to subinhibitory concentrations of colistin (1 µg/ml) observed in this study could be explained as a bacterial adaptive survival response to antibiotic stress^21^. These findings are also consistent with previous research which indicated an increase in development of MDR *Acinetobacter baumanii* biofilms by sub-inhibitory doses of colistin and polymyxin (1/2 and 1/4 MIC) through regulating efflux pump and biofilm-related gene expression^22^. Because antibiotics at sub-inhibitory doses can facilitate biofilm formation in clinically important pathogens through different strategies^21^, it is valuable to further investigate the mechanisms involved in increasing colistin-resistant *K. pneumoniae* biofilms in the presence of antibiotic stress.

Although colistin displayed antibiofilm effects on metabolically inactive cells in the inner layers of *Escherichia coli* and MRSA biofilms with intrinsic colistin resistance, regrowth of colistin-resistant phenotypes was also observed following treatment with 16 mg/L of colistin monotherapy^14,15^. Even though EDTA produced potent antimicrobial activities in reducing biofilm biovolume and biofilm-embedded viable cells, incomplete biofilm eradication and a sharp increase in biofilm cells after 24-h EDTA (12 mg/ml) treatment suggests that antibiotics should be administered in conjunction with EDTA for curative treatment and preventative measures for recurrence of catheter-related biofilm infections. Interestingly, the colistin-EDTA combination displayed improved antibiofilm activities in completely eradicating mature biofilm and inhibiting biofilm bacterial viabilities within 24 h of treatment. This may have occurred due to the biofilm matrix disruption by EDTA's metal ion chelation, which may not only facilitate colistin penetration into biofilm inner layers, but also increase bacterial release, where they act synergistically to produce lethal effects on released biofilm bacteria^9,10,14,23^. Previous studies also demonstrated that EDTA increased bactericidal effects of gentamicin on *Enterobacteriaceae* biofilms by matrix disruption and synergistic bactericidal effects^11^.

To further confirm the efficacy of the colistin-EDTA combination, a catheter-related biofilm infection was developed in immunocompromised mice to obtain a clinically relevant animal model that relates to patients’ conditions commonly seen in clinical settings. Interestingly, administration of colistin-EDTA combination within 24-h exposure not only successfully eradicated catheter-related biofilm infections, but also decreased bacterial viabilities, demonstrating their potent efficacy for eliminating the risk of recurrence both in vitro and in vivo. These findings support the previous study demonstrating the in vitro and in vivo effectiveness of gentamicin-EDTA combination for the eradication of catheter-associated Gram-negative pathogens in Totally Implantable Venous-Access Ports (TIVAP) catheters^11,12^.

Furthermore, the colistin-EDTA combination was observed to reduce bacterial load in internal organs, decrease serum creatinine, and provide significant protection against mortality in treated mice, indicating their significant synergistic therapeutic efficacy and safety in vivo. These results are consistent with previous study in which EDTA enhanced imipenem susceptibilities to treat New Delhi metallo-ß-lactamase-1(NDM 1) producing *E. coli* and reduced systemic bacterial burden in a murine sepsis model^24^. In pneumonic mice induced by *Pseudomonas aeruginosa*, reduced pulmonary bacterial burden and striking improvement with 100% survival were observed after subcutaneous and intranasal administration of imipenem-EDTA combination^25^. Colistin exposure has been linked to increased serum creatinine in treated patients due to their induced oxidative stress in proximal renal tubules^26^. However, the lower dose of colistin in the colistin-EDTA combination, as well as the combined vasodilatation and antioxidant effects of EDTA^27^, may help to mitigate these potential side-effects of colistin in treated mice. Whereas a subinhibitory dose of colistin in colistin-EDTA combination has been shown to prevent bacterial regrowth and achieve significant in vitro and in vivo efficacies, using colistin-EDTA combination could prevent not only the resurgence of the resistant population, but also development of renal complications which are major limiting factors for colistin usage in clinical settings^28^. Although pseudo-thrombocytopenia can occur by flushing or diffusion into the systemic circulation when EDTA is used inside catheters as lock solution^29^, the dose of EDTA used in this study was 12 mg/ml, which is lower than the FDA-approved dose of EDTA for use in lead poisoning (total daily dose of 1000 mg/m^2^)^30^, the dose used in hemodialysis catheters (30 mg/ml)^31^ and slightly higher than the dose for reducing cardiovascular events in diabetic patients with peripheral vascular disease (6 mg/ml)^32^.

This study also revealed altered in vivo expression of various virulence genes of *K. pneumoniae* which include those related to lipopolysaccharide (*wabG* and *uge*) that protect bacteria from host immune defenses for enhancing pathogenicity, porin genes (*ompK* 35 and 36) for nutrient transport to improve bacterial survival inside the host, iron acquisition system genes (*kfu* and *ybtS*) which modulate host immune responses for systemic survival and dissemination, type 3 fimbria adhesin *– mrkD* for bacterial binding to develop biofilm on abiotic surfaces such as catheters, and type 2 quorum-sensing regulatory system gene – *luxS* to promote biofilm development by facilitating cell to cell communication^33^.

. Exposure to antimicrobial agents create a stressful environment for bacteria and it could result in altered expression of bacterial genes which reflects how the bacteria deal with the stress inside the host^34^. Altered in vivo expression of virulence genes found in this study point to the bacterial adaptive response to survive in hostile environment that occurred as the impacts of different treatments tested in mice^35,36^. Given the scarcity of knowledge on the involvement of these genes in the response to various stresses induced by colistin and EDTA treatment in *K. pneumoniae*, further studies are needed to investigate the consequence of virulence gene upregulation following this treatment.

Although the subcutaneous catheter-related biofilm infection model used in this study is relevant to conditions seen in clinical settings and linked to device-associated infections or catheter-associated bloodstream infection, randomized control trials will be required to validate the clinical safety and efficacy of colistin-EDTA combination therapy in the treatment of catheter-related biofilm infection.

In conclusion, this is the first in vitro and in vivo study which highlights a novel colistin-EDTA combination therapy successfully reversing colistin resistance and eradicating *K. pneumoniae* catheter-related biofilm infections, while also demonstrating a favorable safety profile with low resistance and toxicity risks.

## Material and methods

### Strains and growth conditions

The colistin-resistant *K. pneumoniae* clinical isolates (n = 47) with different resistance profiles were obtained without preference from a strain repository at the Department of Microbiology, King Chulalongkorn Memorial Hospital. Clinical strains had been isolated during 2016–2019 from 47 infected patients as part of the standard care of the patients and was not related to the present study. Strains were stored in a repository collection after standard characterization and identification (including 16S rRNA sequencing using primers as documented previously) (Supplementary Table S4). The clinical isolates were cultured on Müller–Hinton agar (Sigma-Aldrich) plates at 37 °C. The strains were stored at − 80 °C in tryptic soy broth (Sigma-Aldrich) with 15% glycerol until they were used in subsequent experiments.

### Antimicrobial agents

Colistin and EDTA were purchased from Sigma-Aldrich and stock solutions were prepared less than 24 h before use. Colistin and EDTA were dissolved in cation-adjusted Müller-Hinton II broth (MHIIB) (Becton Dickinson) medium and the supplemented medium sterilized by filtration through a membrane filter nominally with 0.22 μm pores. Serial dilutions of the colistin and EDTA stocks were prepared in MHIIB medium immediately before use.

### *K. pneumoniae* susceptibilities to colistin and EDTA

*K. pneumoniae* planktonic susceptibility (Minimal Inhibitory Concentrations, MICs) to colistin were established using standard techniques (broth microdilution) according to EUCAST (criteria for *Enterobacteriaceae* for colistin only)^37^and CLSI guidelines^38^. *E. coli* ATCC 25922, and *P. aeruginosa* ATCC 27853 were used as quality control strains. To establish *K. pneumoniae* planktonic MIC for colistin and EDTA, drugs were serially diluted two-fold in 96-well microtiter plates and bacteria added. The MIC to EDTA was also determined by serial dilution as above. The plates were incubated at 37 °C for 18 h.

### Synergistic activities of colistin-EDTA combination against planktonic colistin-resistant *K. pneumoniae* clinical isolates

The synergistic activities of colistin and EDTA combination against planktonic colistin-resistant *K. pneumoniae* clinical isolates were screened by checkerboard assay. The synergistic activities were interpreted as follows: Synergy: FIC index ≤ 0.5; Additive: 0.5 > FIC index ≤ 1; Indifference: 1 > FIC index ≤ 4, and Antagonism: FIC index > 4, respectively^39^.

### Time-kill assay

Planktonic colistin-resistant *K. pneumoniae* isolates that showed the synergistic activities with colistin-EDTA combination were confirmed by using time-kill assay. The bacteria were grown with no drug, each drug, and drug combinations. The viable bacterial cells were collected at 0, 2, 4, 6, 8, 12, and 24 h. After incubation, the CFU/ml of viable bacterial cells were quantified by colony counting on solid media. The synergistic activities were interpreted when there was ≥ 2 log10 (CFU/ml)-fold decrease in combination compared with the single antibiotic; Bactericidal activity was defined as a ≥ 3 log10(CFU/ml)-fold decrease when compared to the number of viable cells at initial time point^39^.

### Quantification of biofilm by crystal violet assay

Quantification of biofilm formation was done by crystal violet assay as described previously^40^. Briefly overnight culture of bacteria were standardized with OD of 0.02 at 600 nm (5 × 10^7^ CFU mL^−1^) and 100 μL aliquots are added in triplicate to flat-bottomed 96-well polystyrene microtiter plates (SPL Life Sciences). The plates were incubated at 37 °C for 24 h. Adherent biofilms were fixed with crystal violet (0.1%) and stained biofilms were solubilized with 30% acetic acid. The absorbance (OD) at 560 nm were determined using a microtiter-plate-reading fluorimeter (Varioskan Flash Multimode Reader; Thermo Fisher Scientific). All experiments were performed in triplicate and repeated three times.

### Effects of colistin, EDTA and colistin-EDTA combination for eradication of in vitro biofilms

The eradication effects of colistin, EDTA, and colistin-EDTA combination on 24 h-old in vitro biofilms of colistin-resistant *K. pneumoniae* were studied to determine the minimum biofilm eradication concentration (MBECs) as described previously^41^. Tests covered single, or combination of colistin- EDTA at 37 °C for the following treatment times; 6, 12, 24, and 48 h. The eradication effects on in vitro biofilms biovolume and biofilm cell viability were analyzed by crystal violet and PrestoBlue assays as described previously^42^.

### Animal study

Female 8-week-old C57BL/6 background mice were purchased from Nomura Siam International (Pathumwan, Bangkok, Thailand) and were used in all experiments. Animals were at rest for 1 week in the animal facility before use. Animals received food and water ad libitum and were housed at a maximum of 2 mice per cage, weighed and closely monitored for any signs of distress throughout experimental periods.

### Catheter-related biofilm infection mouse model

The previously described in vivo catheter-related infection model was performed with modifications^43^ (Fig. 5a). Mice were immunosuppressed by subcutaneous administration of dexamethasone at 0.25 mg/kg/dose once a day for 3 days before catheter implantation, which was maintained throughout the study. To avoid any bacterial contamination of the host, the drinking water was supplemented with ampicillin sodium powder (0.5 g/L) before catheter implantation and stopped one day before implantation. 25-mm catheters (NIPRO) with an inner diameter of 1.45 mm were pre-coated overnight with 1.8 ml of 100% fetal bovine serum (Gibco™; Waltham, MA) at 37° C. Catheters were then inoculated with cell suspensions of colistin-resistant *K. pneumoniae* in MHIIB at final concentrations of 5 × 10^7^ CFU ml^−1^ for 90 min at 37 °C to allow for microbial adhesion to catheters. For each experimental set, in vitro-infected catheters were processed for assessment of microbial recovery to confirm standardized microbial adherence to catheters prior to implantation. Following in vitro microbial adhesion, catheter pieces were rinsed with PBS and kept on ice until implanted. Subcutaneous implantation of catheter pieces (approximately 0.5–1 cm) was done on the left and right side of the flank of animal under isoflurane anesthesia. The incisions were closed with sutures. The wound was disinfected with 0.5% chlorhexidine in 70% alcohol, or with 1% iodine isopropanol. Animals were monitored until they recovered from anesthesia, and then daily for any developing clinical signs of distress. Animals were euthanized by isoflurane inhalation and collected samples (catheters, blood, heart, tissues surrounding catheter, lungs, spleen, and kidneys) were aseptically harvested individually.

### Effects of colistin, EDTA and colistin-EDTA combination on catheter-related biofilm infection mouse model

Animals were divided into control (no therapy) group and experimental (therapy) group with 3 subgroups. The catheter-related biofilm infections of colistin-resistant *K. pneumoniae* in implanted catheters were exposed to PBS (control), colistin (1 µg/ml), EDTA (12 mg/ml) and colistin- EDTA (1 µg/ml + 12 mg/ml) for a total of 4 groups with 10 animals in each group. After 24 h of different treatments, catheters were removed under aseptic conditions and the efficacies of each treatment in different groups were analyzed by confocal laser scanning microscope (biomass, Live/Dead ratio and biovolume inhibition) and bacterial counting^44^. In addition, animals were monitored regularly for 7 days or until death, whichever occurred first, to determine their survival. Also, mice were sacrificed on different days post-infection by cervical dislocation to determine viable cell count (CFU/ml) in treated catheter and internal organs (blood, heart, kidneys, lungs, spleen, and tissues surrounding catheter) after each treatment as described previously^24^. Measurements of serum creatinine levels were done after exposure to each treatment in all treated groups as described previously^45^.

### Confocal laser scanning microscopic analysis (CLSM)

For CLSM analysis, supernatants are carefully removed and biofilms are subsequently stained with the LIVE/DEAD Bacterial Viability Kit (Invitrogen) according to the manufacturer’s protocol. MATLAB-based tool PHLIP (without connected volume filtration) were used to calculate descriptive parameters of biofilms (including biovolume, substratum coverage, area-to-volume ratio, spatial spreading and 3D colocalization) from the integrated total of each individual slice of a thresholded z-stack as described previously^42,46,47^. The calculation of the different proportions of green (live bacteria) as well as red and yellow/colocalized (dead bacteria) biovolumes from the analyzed stacks were using the 'colocalization in 3D' value and the parameters 'red', 'green', and 'total biovolume' (in μm^3^) generated by the PHLIP software as described previously^47,48^. A biofilm was considered affected by an antibiotic or EDTA or combination within the given concentration range when there is a constant increase in the red + yellow (RY) biovolume fraction within the given antibiotic concentration range and this fraction is at least 80% of the total biovolume.

### Effect of single and combination of colistin-EDTA on in vivo virulence gene expression

The expression level of virulence factors (*kfu luxS, mrkD, ompK35, ompK36, uge, wabG* and *ybtS*) were determined with Quantitative RT-PCR (qRT-PCR) using the specific primers as described previously^48,49^. All primers are listed in Supplementary Table S4.

Total mRNA were extracted from control and experimental groups of in vivo catheter-related biofilm infection that were challenged with various treatments for 24 h. All samples are analyzed in triplicate. Using the housekeeping gene—16srRNA for normalization, the relative quantification of gene expression for each treatment group was computed using the ΔΔ^CT^ (CT is threshold cycle) method to evaluate and compare fold change differences.

### Data analysis

All statistical analysis was conducted using R statistic package^50^. The data were compared by either unpaired two-tailed Student’s t-test or unpaired two-tailed Mann–Whitney’s U test. All data were presented as the mean ± s.d. Statistical significance was accepted at *p* < 0.05, *p* < 0.01, *p* < 0.001, and *p* < 0.0001.

### Ethical approval

The study protocol was approved by the Institutional Review Board (IRB) of the Faculty of Medicine, Chulalongkorn University, Bangkok, Thailand (COA No. 045/2020, IRB No. 774/63) was performed in accordance with the ethical standards as laid down in the 1964 Declaration of Helsinki and its later amendments and comparable ethical standards. Animal care and use protocol are based upon the National Institutes of Health (NIH), USA. The protocol was approved by the Institutional Animal Care and Use Committee of the Faculty of Medicine, Chulalongkorn University, Bangkok, Thailand (Certificate No- 033/2563, Research Project No. – 020/2563). The study was carried out in compliance with the ARRIVE guidelines (Animal Research: Reporting of In Vivo Experiments).

### Informed consent

For this retrospective study of anonymous clinical isolates, the requirement for informed consent from patients was waived by Institutional Review Board (IRB) of the Faculty of Medicine, Chulalongkorn University, Bangkok, Thailand (COA No. 045/2020, IRB No. 774/63).

## Supplementary Information

Supplementary Information.

## Data Availability

The authors confirm that the data supporting the findings of this study are available within the article and its additional information.
