# Supplementary material for: Novel colistin-EDTA combination for successful eradication of colistin-resistant Klebsiella pneumoniae catheter-related biofilm infections
Source: Sci Rep. 2021 Nov 4;11:21676. doi: 10.1038/s41598-021-01052-5 (PMC8568960; doi:10.1038/s41598-021-01052-5)
Supplement: Supplementary file 1 — Supplementary Information. [file 41598_2021_1052_MOESM1_ESM.docx]

**Novel colistin-EDTA combination for successful eradication of colistin-resistant *Klebsiella pneumoniae* catheter-related biofilm infections**

Aye Mya Sithu Shein^1,2,3,†^, Dhammika Leshan Wannigama^1,2,4, †^, Paul G. Higgins^5,6,7, †^,Cameron Hurst^8, †^, Shuichi Abe^9, †^, Parichart Hongsing^10,11, †^, Naphat Chantaravisoot^12,13^,Thammakorn Saethang^14^, Sirirat Luk-in^15^, Tingting Liao ^16,17^, Sumanee Nilgate^1,2^, Ubolrat Rirerm^1,2^, Naris Kueakulpattana ^1,2^, Matchima Laowansiri^1,2^, Sukrit Srisakul^1,2^, Netchanok Muhummudaree^1,2^, Teerasit Techawiwattanaboon ^1, 18^, Lin Gan^19^, Chenchen Xu^20^, Rosalyn Kupwiwat^21^, Phatthranit Phattharapornjaroen^22,23^, Rojrit Rojanathanes ^24^, Asada Leelahavanichkul^1,25^, Tanittha Chatsuwan^1,2,*^

^1^ Department of Microbiology, Faculty of Medicine, Chulalongkorn University, King Chulalongkorn Memorial Hospital, Thai Red Cross Society, Bangkok, Thailand.

^2^ Antimicrobial Resistance and Stewardship Research Unit, Faculty of Medicine, Chulalongkorn University, Bangkok, Thailand.

^3^​Interdisciplinary Program of Medical Microbiology, Graduate School, Chulalongkorn University, Bangkok, Thailand.

^4^ School of Medicine, Faculty of Health and Medical Sciences, The University of Western Australia, Nedlands, Western Australia, Australia.

^5^ Institute for Medical Microbiology, Immunology and Hygiene, Faculty of Medicine and University Hospital Cologne, University of Cologne, Cologne, Germany.

^6^Center for Molecular Medicine Cologne, University of Cologne, Faculty of Medicine and University Hospital Cologne, Cologne, Germany

^7^ German Centre for Infection Research, Partner site Bonn-Cologne, Cologne, Germany.

^8^ Statistics, QIMR Berghofer Medical Research Institute, Brisbane, Queensland, Australia.

^9^ Department of Infectious Diseases and Infection Control, Yamagata Prefectural Central Hospital, Yamagata, Japan.

^10^ Mae Fah Luang University Hospital, Chiang Rai, Thailand.

^11^ School of Integrative Medicine, Mae Fah Luang University, Chiang Rai, Thailand.

^12^ Department of Biochemistry, Faculty of Medicine, Chulalongkorn University, Bangkok, Thailand.

^13^ Center of Excellence in Systems Biology, Research Affairs, Faculty of Medicine, Chulalongkorn University, Bangkok, Thailand.

^14^ Department of Computer Science, Faculty of Science, Kasetsart University, Bangkok, Thailand.

^15^ Department of Clinical Microbiology and Applied Technology, Faculty of Medical Technology, Mahidol University, Bangkok, Thailand.

^16^ Department of Physiology, Faculty of Medicine, Chulalongkorn University, Bangkok, Thailand.

^17^ Center of Excellence for Microcirculation, Faculty of Medicine, Chulalongkorn University

^18^ Chula Vaccine Research Center, Faculty of Medicine, Chulalongkorn University, Bangkok, Thailand

^19^ Department of General surgery, Fuling Center Hospital of Chongqing City, Chongqing, China

^20^ In-patient pharmacy, Jiangsu Province Hospital, The First Affiliated Hospital of Nanjing Medical University, Nanjing, China

^21^ Chulabhorn International College of Medicine, Thammasat University, Thammasat University Hospital, Bangkok, Thailand.

^22^ Department of Emergency Medicine, Center of Excellence, Faculty of Medicine Ramathibodi Hospital, Mahidol University, Bangkok, Thailand

^23^ Institute of Clinical Sciences, Department of Surgery, Sahlgrenska Academy, Gothenburg University, 40530 Gothenburg, Sweden

^24^ Center of Excellence in Materials and Bio-Interfaces, Faculty of Science, Chulalongkorn University, Bangkok, Thailand

^25^ Translational Research in Inflammation and Immunology Research Unit (TRIRU), Department of Microbiology, Chulalongkorn University, Bangkok, Thailand

^†^These authors contributed equally to this work as first authors

**Supplementary information**

**Supplementary Table S 1.**

Minimal inhibitory concentrations (MIC, μg/ml) of different antimicrobial agents against 47 colistin-resistant *K. pneumoniae* clinical isolates

**Supplementary Table S 2.**

Antibiotic susceptibility patterns of 47 colistin-resistant *K. pneumoniae* clinical isolates.

**Supplementary Table S 3.**

Crystal Violet assay for quantification of *in vivo* colistin-resistant *K. pneumoniae* biofilms

**Supplementary Table S 4.** Primers used in this study.

**Supplementary Table S 1.**

| Specimens | Minimal inhibitory concentrations (MIC, μg/ml) of different antimicrobial agents | | | | | | | |
| --- | --- | --- | --- | --- | --- | --- | --- | --- |
|  | Colistin | Imipenem | Meropenem | Ceftazidime | Ciprofloxacin | Amikacin | Fosfomycin | EDTA |
| Extensively  drug-resistant (XDR)  (n=43) | 8 - >2048 | 1 - >512 | 0.25 - >512 | 128 ->512 | 32- >512 | 2 - >512 | 8 - >512 | 3 - 24 |
| Pandrug-resistant (PDR)  (n=4) | 16-32 | 8-256 | 64-256 | >512 | 64 - >512 | >512 | 256 - >512 | 12 - 24 |

**Supplementary Table S 2.**

| Antimicrobial agents | Antibiotic susceptibility patterns | | |
| --- | --- | --- | --- |
|  | Resistance (%) | Intermediate (%) | Sensitive (%) |
| Colistin | 100% | - | - |
| Imipenem | 95.75% | - | 4.25% |
| Meropenem | 95.74% | - | 4.25% |
| Ceftazidime | 100% | - | - |
| Ciprofloxacin | 100% | - | - |
| Amikacin | 23.41% | 31.91% | 44.68% |
| Fosfomycin | 57.46% | 14.89% | 27.65% |

| Specimens | Crystal Violet assay (OD 560) | | | | | | | |
| --- | --- | --- | --- | --- | --- | --- | --- | --- |
|  | 1 Day | 2 Days | 3 Days | 4 Days | 5 Days | 6 Days | 7 Days | 8 Days |
| Pandrug-resistant  (PDR) | 1.61 | 1.89 | 2.485 | 2.83 | 3.24 | 3.82 | 4.89 | 4.97 |
| Extensively drug-resistant (XDR) | 1.01 | 1.38 | 1.88 | 2.11 | 3.12 | 3.34 | 4.33 | 4.34 |

**Supplementary Table S 3.**

**Supplementary Table S 4.**

| **Target genes** | **Primers’ sequences (5′ to 3′)** | **Tm (°C)** | **References** |
| --- | --- | --- | --- |
| 16srRNA - for | AGAGTTTGATCCTGGCTCAG | 46 | ^1^ |
| 16srRNA - rev | GGTTACCTTGTTACGACTT |  |  |
| *kfu* - for | GGCCTTTGTCCAGAGCTACG | 60 | ^2^ |
| *kf*u - rev | GGGTCTGGCGCAGAGTATGC |  |  |
| *ybtS* - for | GACGGAAACAGCACGGTAAA |  |  |
| *ybt*S- rev | GAGCATAATAAGGCGAAAGA |  |  |
| *mrk*D - for | AAGCTATCGCTGTACTTCCGGCA |  |  |
| *mrkD* - rev | GGCGTTGGCGCTCAGATAGG |  |  |
| *lux*S - for | AGTGATGCCGGAACGCGG | 53 | ^3^ |
| *luxS* - rev | CGGTGTACCAATCAGGCTC |  |  |
| *ompK35* - for | GCAATATTCTGGCAGTGGTGATC | 51 |  |
| *ompK35* - rev | ACCATTTTTCCATAGAAGTCCAGT |  |  |
| *ompK36* - for | TTAAAGTACTGTCCCTCCTGG | 50 |  |
| *ompK36* - rev | TCAGAGAAGTAGTGCAGACCGTCA |  |  |
| *uge* - for | TCTTCACGCCTTCCTTCACT | 53 | ^4^ |
| *uge* - rev | GATCATCCGGTCTCCCTGTA |  |  |
| *wabG*- for | ACCATCGGCCATTTGATAGA | 51 |  |
| *wabG* - rev | CGGACTGGCAGATCCATATC |  |  |

**References**

1 Srinivasan, R. *et al.* Use of 16S rRNA gene for identification of a broad range of clinically relevant bacterial pathogens. *PLoS One* **10**, e0117617 (2015).

2 Compain, F. *et al.* Multiplex PCR for detection of seven virulence factors and K1/K2 capsular serotypes of *Klebsiella pneumoniae*. *J Clin Microbiol* **52**, 4377-4380 (2014).

3 Vuotto, C. *et al.* Biofilm formation and antibiotic resistance in *Klebsiella pneumoniae* urinary strains. *J Appl Microbiol* **123**, 1003-1018 (2017).

4 Yu, W. L. *et al.* Association between rmpA and magA genes and clinical syndromes caused by Klebsiella pneumoniae in Taiwan. *Clin Infect Dis* **42**, 1351-1358 (2006).
